# Familial co-aggregation of attention-deficit/hyperactivity disorder and autoimmune diseases: a cohort study based on Swedish population-wide registers

**DOI:** 10.1093/ije/dyab151

**Published:** 2021-08-11

**Authors:** Tor-Arne Hegvik, Qi Chen, Ralf Kuja-Halkola, Kari Klungsøyr, Agnieszka Butwicka, Paul Lichtenstein, Catarina Almqvist, Stephen V Faraone, Jan Haavik, Henrik Larsson

**Affiliations:** Department of Biomedicine, University of Bergen, Bergen, Norway; Department of Medical Epidemiology and Biostatistics, Karolinska Institutet, Stockholm, Sweden; Department of Medical Epidemiology and Biostatistics, Karolinska Institutet, Stockholm, Sweden; Department of Medical Epidemiology and Biostatistics, Karolinska Institutet, Stockholm, Sweden; Department of Global Public Health and Primary Care, University of Bergen, Bergen, Norway; Division of Mental and Physical Health, Norwegian Institute of Public Health, Bergen, Norway; Department of Medical Epidemiology and Biostatistics, Karolinska Institutet, Stockholm, Sweden; Department of Child Psychiatry, Medical University of Warsaw, Warsaw, Poland; Department of Medical Epidemiology and Biostatistics, Karolinska Institutet, Stockholm, Sweden; Department of Medical Epidemiology and Biostatistics , Karolinska Institutet, Stockholm, Sweden; Pediatric Allergy and Pulmonology Unit at Astrid Lindgren Children’s Hospital, Karolinska University Hospital, Stockholm, Sweden; Departments of Psychiatry and of Neuroscience and Physiology, SUNY Upstate Medical University, Syracuse, NY, USA; Department of Biomedicine, University of Bergen, Bergen, Norway; Division of Psychiatry, Haukeland University Hospital, Bergen, Norway; Department of Medical Epidemiology and Biostatistics, Karolinska Institutet, Stockholm, Sweden; School of Medical Sciences, Örebro University, Örebro, Sweden

**Keywords:** Attention-deficit/hyperactivity disorder, neurodevelopmental disorder, autoimmunity, familial aggregation, familial co-aggregation, genetics, cohort study, national register, maternal immune activation

## Abstract

**Background:**

Attention-deficit/hyperactivity disorder (ADHD) has been associated with several autoimmune diseases (AD), both within individuals and across relatives, implying common underlying genetic or environmental factors in line with studies indicating that immunological mechanisms are key to brain development. To further elucidate the relationship between ADHD and autoimmunity we performed a population-wide familial co-aggregation study.

**Methods:**

We linked Swedish national registries, defined a birth cohort with their biological relatives and identified individuals diagnosed with ADHD and/or 13 ADs. The cohort included 5 178 225 individuals born between 1960 and 2010, of whom 118 927 (2.30%) had been diagnosed with ADHD. We then investigated the associations between ADHD and ADs within individuals and across relatives, with logistic regression and structural equation modelling.

**Results:**

Within individuals, ADHD was associated with a diagnosis of any of the 13 investigated ADs (adjusted odds ratio (OR) =1.34, 95% confidence interval (CI) = 1.30-1.38) as well as several specific ADs. Familial co-aggregation was observed. For example, ADHD was associated with any of the 13 ADs in mothers (OR = 1.29, 95% CI = 1.26–1.32), fathers (OR = 1.14, 95% CI = 1.11–1.18), full siblings (OR = 1.19, 95% CI = 1.15–1.22), aunts (OR = 1.12, 95% CI = 1.10–1.15), uncles (OR = 1.07, 95% CI = 1.05–1.10) and cousins (OR = 1.04, 95% CI = 1.03–1.06). Still, the absolute risks of AD among those with ADHD were low. The genetic correlation between ADHD and a diagnosis of any of the investigated ADs was 0.13 (95% CI = 0.09–0.17) and the environmental correlation was 0.02 (95% CI = -0.03–0.06).

**Conclusions:**

We found that ADHD and ADs co-aggregate among biological relatives, indicating that the relationship between ADHD and autoimmune diseases may in part be explained by shared genetic risk factors. The patterns of familial co-aggregation of ADHD and ADs do not readily support a role of maternal immune activation in the aetiology of ADHD. The findings have implications for aetiological models of ADHD. However, screening for autoimmunity among individuals with ADHD is not warranted.

Key Messages

- Some studies show that individuals with attention-deficit/hyperactivity disorder (ADHD), and their biological relatives, have an increased risk of autoimmune disorders, though the aetiology underlying this increased risk remains unclear.
- Using Swedish population-wide registers it was found that individuals diagnosed with ADHD, and their biological relatives, had a moderately increased risk of autoimmune diseases.
- The noted associations between ADHD and autoimmune diseases may in part be explained by shared genetic risk factors.
- Due to the low absolute risks and limited benefit, screening for autoimmunity among individuals with ADHD is not warranted.

## Introduction

Attention-deficit/hyperactivity disorder (ADHD) is a common, and often chronic, childhood-onset neurodevelopmental disorder with hyperactivity, inattention and impulsivity constituting the core symptoms.^1^ Despite its strong familial aggregation (e.g. an individual has an over seven times increased risk of being diagnosed with ADHD if a full sibling has ADHD), a high heritability of about 70–80% and several recently identified genome-wide statistically significant genetic loci, the precise aetiology of ADHD remains elusive.^1–5^

Several immunological mechanisms are central to brain development and functioning, e.g. the primary immune cells of the brain, glial cells, are key to synaptic pruning during neurodevelopment,^6^ and various immune signalling molecules, such as cytokines and complement factors, have been demonstrated to regulate and affect neurogenesis.^7–9^ Traits and disorders characterized by immune dysfunction could therefore be associated with neurocognitive and behavioural traits and disorders, reflecting shared aetiology. In line with this, immune-mediated comorbidities of ADHD have in recent years received increased attention^10^^,^^11^ and common immune-mediated diseases such as asthma and eczema have repeatedly been linked to ADHD.^12–15^ Similarly, ADHD has been related to autoimmune diseases (ADs), with epidemiological studies reporting positive within-individual associations with several specific ADs, such as celiac disease, ulcerative colitis, psoriasis, ankylosing spondylitis and type 1 diabetes mellitus (T1DM), in addition to a general association with ADs as a group of diseases.^12^^,^^16–19^ Although such findings are intriguing, many have not been replicated in independent samples.^10^^,^^11^^,^^17^ Some studies also suggest that ADHD might co-aggregate with ADs within families.^18^^,^^20^^,^^21^ Such findings could suggest genetic sharing between ADHD and ADs, though none have investigated whether the familial clustering extends beyond first-degree relatives. Moreover, genetic studies have reported contrasting results. For example, a study that utsed data from genome-wide association studies (GWAS) reported a negative genetic correlation between ADHD and T1DM.^2^^,^^22^ Consequently, alternative mechanisms must be considered. It could be that the environment shared between-first degree relatives may have a role in the familial co-aggregation of ADHD and ADs; for example, socioeconomic status, pollution and exposure to infectious agents have all been associated with both ADHD and ADs.^23–25^ Moreover, in light of animal studies that have established gestational maternal inflammation as a potential cause of offspring neurodevelopmental disorders^8^^,^^26^ and the findings of stronger association between offspring ADHD and maternal ADs as compared with offspring ADHD and paternal ADs,^18^^,^^20^^,^^21^ AD-mediated maternal effects are conceivable.

To further advance knowledge on the relationship between ADHD and ADs, we conducted a population-based, within-individual-and familial, co-aggregation cohort study using Swedish population-wide national registers. If ADHD was found to be associated with ADs across relatives who are assumed to share little to no environment, such as cousins, this would be in support of common genetic factors. Moreover, if one saw stronger associations between ADHD and ADs across maternal relatives, as compared with paternal relatives, this could indicate a role of AD-mediated maternal effects.

## Methods

The study was approved by the Regional Ethical Review Board in Stockholm, Sweden (reference number 2013/862–31/5). As the study is register-based, informed consent was waived.

### Study population

The present cohort study was based on linked data from several Swedish population-based registers, using each individual’s unique national personal identification number for linkage.^27^ With the Swedish Total Population Register we identified all 5 403 464 individuals born in Sweden between 1960 and 2010, and then restricted to the 5 388 383 individuals where the identity of the biological mother was known.^28^ By using the Swedish Cause of Death Register and the Total Population Register,^28^^,^^29^ we excluded all 210 158 individuals who died or emigrated before age 10, leaving us with a cohort of 5 178 225 index individuals.

**Figure 1.**
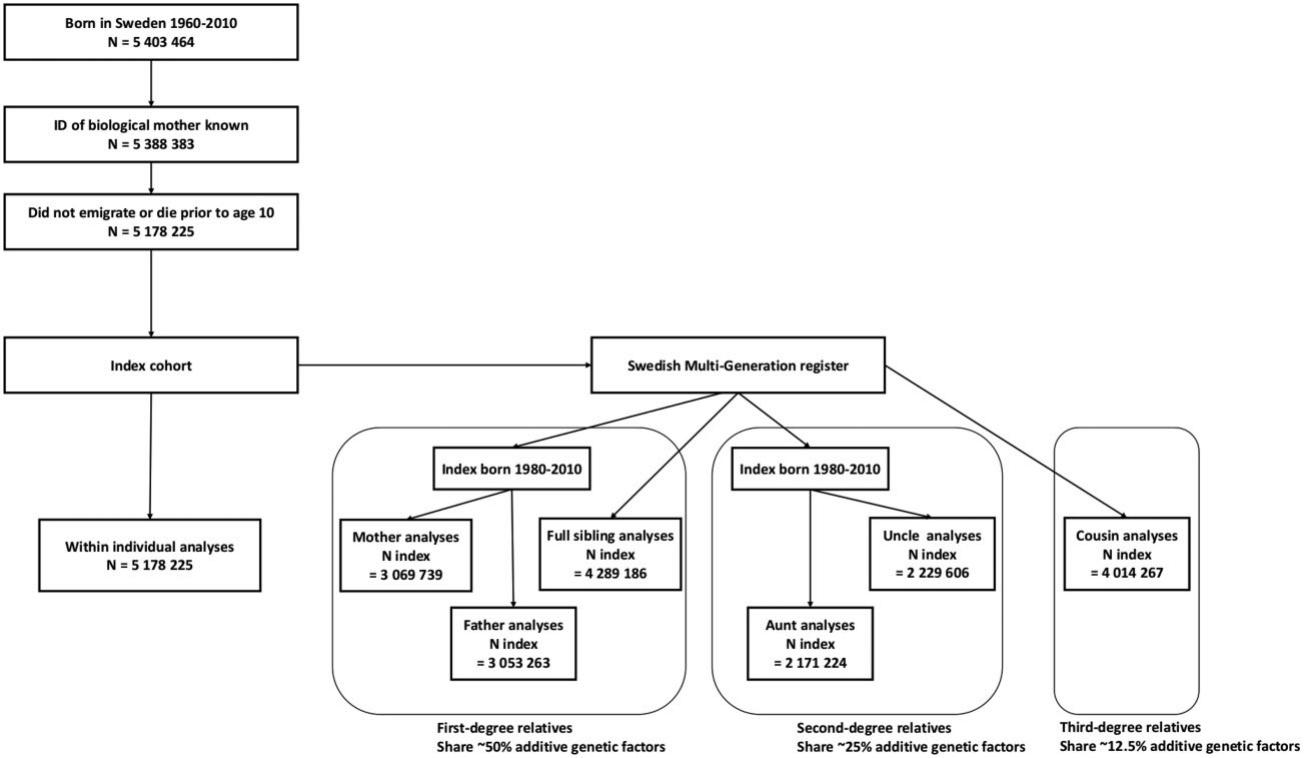
Flowchart illustrating how the study population was defined

The Swedish Multi-Generation Register contains information on the biological parents of Swedish inhabitants born after 1931 and alive in Sweden after 1960.^30^ With this register we deduced family relationships and constructed several index-relative cohorts: mothers, fathers, full siblings, aunts, uncles and cousins. To limit bias due to mortality in the parent generation, we restricted the index-relative cohorts with mothers, fathers, aunts and uncles to index individuals born in 1980 or later. We excluded 60 025 siblings, 18 798 aunts, 23 169 uncles and 93 797 cousins who died or emigrated before age 10 from all analyses. For the total cohort sizes and analytical samples see Table 1. See Figure 1 for a flowchart that describes the definition of the study population.

**Table 1:** Number of index individuals, relatives and unique index-relative pairs in the investigated cohorts

Cohort	Number of unique index individuals	Number of unique relatives	Number of unique index-relative pairs	Estimated additive genetic factors shared
Within-individual^a^	5 178 225			
Index-mother^b^	3 069 739	1 586 303	3 069 739	50%
Index-father^b^	3 053 263	1 579 362	3 053 263	50%
Index-full sibling^a^	4 289 186	4 592 004	3 911 001	50%
Index-aunt^b^	2 171 224	1 406 855	4 202 774	25%
Index-uncle^b^	2 229 606	1 483 983	4 439 571	25%
Index-cousin^a^	4 014 267	4 276 679	13 869 192	12.5%

a Index individuals born 1960–2010.

b Index individuals born 1980–2010.

### Ascertainment of ADHD and autoimmune diseases

The Swedish National Patient Register (NPR) was established in 1964 as a somatic inpatient care register.^31^ From 1973 it has included psychiatric inpatient care, and since 2001 somatic and psychiatric outpatient care. Diseases and disorders recorded in the NPR have been coded according to Swedish adaptations of the World Health Organization’s International Classification of Disease (ICD); ICD-7 from 1964 to 1967, ICD-8 from 1968 to 1986, ICD-9 from 1987 to 1996 and ICD-10 from 1997.

The Swedish Prescribed Drug Register (PDR) was established July 2005 and holds information on all dispensed drug prescriptions in Sweden.^32^ Specific drugs are recorded with their Anatomical Therapeutic Chemical (ATC) code. Over-the-counter drugs are not recorded. We used information on dispensed drugs from July 2005 to December 2013.

We identified individuals ever diagnosed with ADHD and ADs of interest using NPR and PDR. We only included ADs with more than 2000 diagnosed individuals in the index cohort. Since ADs share genetic architecture, and one individual might have several ADs, we defined an ‘any of the 13 ADs’ variable (anyAD) that constituted our primary outcome; see Supplementary Table S1 (available as Supplementary data at *IJE* online) for case definitions.

### Data management and statistical analyses

We organized, managed and analysed the data with Statistical Analysis Software (SAS) 9.4 (SAS Institute Inc.) and R 3.4.3 (R Foundation) with built-in commands, and the drgee,^33^ stdReg^34^ and OpenMx^35^ packages.

Associations between ADHD and ADs were calculated as odds ratios (OR) with logistic regression. In all analyses, lifetime ADHD defined predictor/exposure and the dependents/outcomes were defined as lifetime diagnosis of the respective ADs, with anyAD being the primary outcome and the specific ADs secondary outcomes. To adjust for familial clustering, we computed cluster-robust standard errors; 95% confidence intervals (95% CI) were calculated *ad modum* Wald. We did not adjust for multiple testing, given that tests across different index-relatives relationships would not constitute independent tests.

Absolute estimates, such as absolute risk (AR) and risk difference (RD), may be more informative in clinical and public health settings than relative measures, such as ORs.^36^ We therefore used logistic regression models and regression standardization approaches to calculate estimates of standardized ARs and RDs.^34^ For the sake of brevity and comparability, we mainly present ORs.

### Within-individual and familial co-aggregation analyses

We calculated the associations between ADHD and ADs within individuals with adjustment for sex and year of birth. Year of birth was modelled as natural cubic splines to minimize residual confounding and loss of statistical power.^37^ We did not adjust for other covariates that are often adjusted for (e.g. maternal education, family income, parental psychiatric disorder, parental AD) as these are likely not true confounders of the association between ADHD and AD, but may rather represent either mediators between ADHD and ADs, or proxies of ADHD and/or AD risk or alternatively proxies for the associations we aim to measure.^2^^,^^38^ To assess sex effects we refitted the models with the addition of an interaction term between ADHD and sex, and we performed the analyses stratified by sex.

In the familial co-aggregation analyses we investigated whether the relatives of individuals diagnosed with ADHD had an increased odds of ADs, as compared with the relatives of non-ADHD individuals. The analyses were adjusted for year of birth of both the index individuals and the relatives modelled as natural cubic splines. Other covariates were not adjusted for under the same rationales as in the within-individual analyses.

Differences in the ORs for the associations between index (offspring) ADHD and maternal AD as compared with index ADHD and paternal AD, could be due to maternal (including gestational) effects. To test whether the index-mother associations were different from the index-father associations, we conducted a Wald test: we merged the mother and father cohorts and conducted ‘parent analyses’ with the inclusion of an interaction term between parent type (mother or father), and ADHD (and all covariates). The resulting estimate for the interaction may thus reflect a maternal effect. As reference, we performed similar analyses for: ‘aunts versus uncles’ unconditioned by parental side, where we assumed no maternal effects; and ‘maternal aunts and uncles versus paternal aunts and uncles’, where we assumed that ADs on the maternal side would be associated with maternal autoimmunity, and thus indirectly with maternal effects on the offspring.

### Sensitivity analyses

We conducted several sensitivity analyses to investigate whether the results in the main analyses could be due to the direct effects of ADHD on ADs, direct effects of ADs on ADHD, the psychiatric comorbidities of ADHD, off-label prescription of ADHD medication in the treatment of ADs, misdiagnosis (including psychosomatic disorders and other AD mimics), geographical factors, period effects related to the registers (including immortal time bias) and right-censoring; see Supplementary information (available as Supplementary data at *IJE* online) for rationales and methods descriptions.

### Quantitative genetic modelling

To assess the relative contribution of genetic and environmental factors to the association between ADHD and anyAD, we performed structural equation modelling. We used full siblings, assumed to share 50% genetic variance and 100% shared environmental variance, and full cousins, assumed to share 12.5% genetic variance and 0% shared environmental variance. All included individuals were born 1960–2010 and did not die or emigrate before age 10. From each family we randomly selected one pair of siblings or cousins, resulting in 1 663 093 sibling pairs and 107 800 cousin pairs. We decomposed ADHD and anyAD’s variance and covariance into additive genetic (A), non-shared environmental (C) and unique environmental (E) factors, using liability threshold models that again were used to fit bivariate models to calculate the correlation of A, C and E across ADHD and anyAD. The analyses were adjusted for year of birth (modelled as a linear and cubic covariate) and sex. The presented results are from the best-fitting and most parsimonious model (lowest Akaike information criteria).

## Results

### Descriptive statistics

Of the 5 178 225 individuals (2 518 377 female, 2 659 848 male) in the final birth cohort, 118 927 (2.3%) were defined as having ADHD. Descriptive information on the composition of the cohorts is presented in Table 1 and information on the prevalences, age distribution and sex distributions of the disorders and diseases of interest is presented in Table 2 and Supplementary Figure S1 (available as Supplementary data at *IJE* online).

**Table 2.** Number and sex distribution per disorder/disease in the index cohort (born 1960–2010)

Disorder/disease	*N* index cohort (prevalence: *N* per 10 000)	Female %	*N* individuals with both ADHD and autoimmune disease
Total	5 178 225	48.6	
ADHD	118 927 (230)	36.0	
Any of the autoimmune diseases	214 596 (414)	56.3	5577
Ankylosing spondylitis	7196 (14)	40.4	138
Celiac disease	37 872 (73)	63.0	1417
Crohn’s disease	21 971 (42)	51.5	548
Grave’s disease	14 111 (27)	83.7	265
Hashimoto’s disease	7548 (15)	84.8	248
Multiple sclerosis^a^	8900 (17)	70.5	88
Psoriasis	45 954 (89)	49.8	1270
Rheumatoid arthritis	15 090 (29)	74.0	255
Sarcoidosis	6960 (13)	37.2	113
Sjögren’s syndrome	2858 (6)	88.6	61
Systemic lupus erythematosus	3452 (7)	87.2	51
Type 1 diabetes mellitus	34 653 (67)	42.9	1081
Ulcerative colitis	30 414 (58)	48.1	606

a Slightly smaller total sample size of 5 160 610 due to case definition.

Abbreviations: ADHD attention-deficit/hyperactivity disorder.

### Within-individual and familial co-aggregation analyses

**Figure 2.**
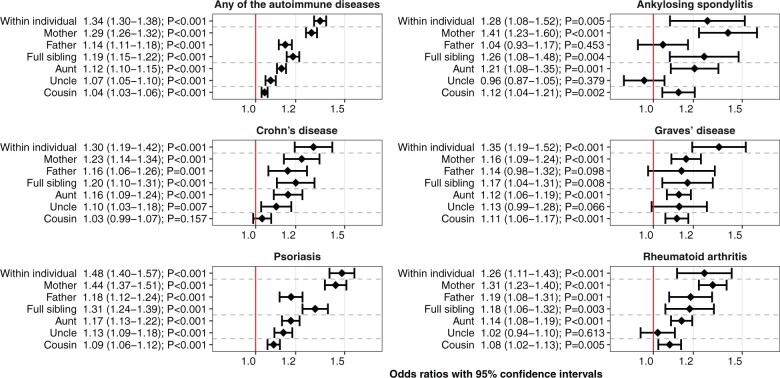
The associations between index individuals’ attention-deficit/hyperactivity disorder (ADHD) and index individuals’ or relatives’ any of the autoimmune diseases (anyAD) and five specific autoimmune diseases, as odds ratios with 95% confidence intervals and *P*-values (adjusted for year of birth of index and relative if applicable). The x-axes are log-transformed

ADHD was associated with anyAD (OR = 1.34, 95% CI = 1.30–1.38) and several specific ADs with ORs ranging between 1.11 (95% CI = 1.02–1.20) for ulcerative colitis and 1.79 (95% CI = 1.38–2.31) for Sjögren's syndrome; see Figure 2 and Supplementary Figure S2 (available as Supplementary data at *IJE* online) for within-individual and familial co-aggregation analyses presented as ORs.

Minor sex differences were noted for ADHD’s association with psoriasis and Hashimoto’s disease; see Supplementary Table S3 (available as Supplementary data at *IJE* online) for sex-specific within-individual results.

In the familial co-aggregation analyses, ADHD was associated with an increased odds of anyAD in all relatives: mothers (OR = 1.29, 95% CI = 1.26–1.32), fathers (OR = 1.14, 95% CI = 1.11–1.18), full siblings (OR = 1.19, 95% CI = 1.15–1.22), aunts (OR = 1.12, 95% CI = 1.10–1.15), uncles (OR = 1.07, 95% CI = 1.05–1.10) and cousins (OR = 1.04, 95% CI = 1.03–1.06). Moreover, ADHD was associated with all specific ADs in at least one of the first-degree relatives (father, mother, full sibling), including all first-degree relatives for Crohn’s disease, psoriasis and rheumatoid arthritis. ADHD was also associated with an increased odds of several specific ADs in aunts, uncles and cousins; see Figure 2 and Supplementary Figure S2 (available as Supplementary data at *IJE* online) for the results of the within-individual and family co-aggregation analyses presented as ORs.

Among individuals diagnosed with ADHD, and their relatives, the standardized ARs for ADs were modestly elevated (Supplementary Figure S3, available as Supplementary data at *IJE* online).

**Figure 3:**
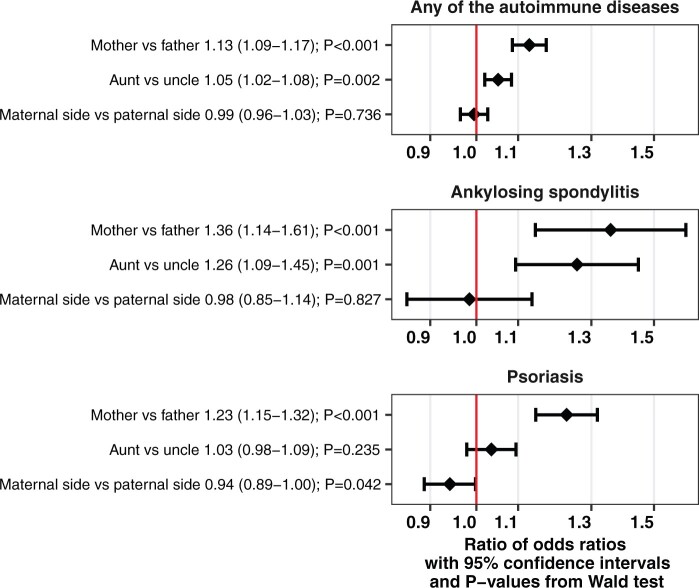
The interaction by parent type on the association between index attention-deficit/hyperactivity disorder (ADHD) and parental any of the autoimmune diseases (anyAD) and two specific autoimmune diseases, as exponentiated coefficients with 95% confidence intervals and *P*-values (adjusted for year of birth of index and relative). The exponentiated coefficient represents the ratio of the two odds ratios (OR), e.g. OR_index-mother_ and OR_index-father_ in the analyses of ‘mother vs father’. If the ratio is >1, it indicates that OR_index-mother_ is higher than OR_index-father_; similarly for ‘aunt vs uncle’, and ‘maternal aunt and uncle vs paternal aunt and uncle’ (maternal side vs paternal side). The x-axes are log-transformed

Index ADHD had stronger association with maternal anyAD as compared with paternal anyAD (ratio of odds ratios (ROR)=1.13, 95% CI = 1.09–1.17). ADHD had stronger association with aunt anyAD, as compared with uncle anyAD (ROR = 1.05, 95% CI = 1.02–1.08). There was no signal for a stronger or weaker association between ADHD and maternal aunt and uncle anyAD as compared with paternal aunt and uncle anyAD (ROR = 0.99, 95% CI = 0.96–1.03). Statistical power was limited in the analyses of the specific ADs; see Figure 3 and Supplementary Figure S4 (available as Supplementary data at *IJE* online).

### Sensitivity analyses

After exclusion of index individuals who had ever received a diagnosis of a non-ADHD psychiatric disorder, the within-individual and index-relative ADHD-AD associations were attenuated and the 95% CIs were widened; see Supplementary information and Supplementary Figure S6 (available as Supplementary data at *IJE* online).

The results of the other sensitivity analyses were not in contrast to the main analyses; see Supplementary information (available as Supplementary data at *IJE* online).

### Quantitative genetic modelling

The best-fitting and most parsimonious model was a model that described ADHD with AE, and anyAD with ACE. As shown in Table 3, the heritability was estimated to 0.84 (95% CI = 0.83–0.86) for ADHD and 0.23 (95%=0.15–0.32) for anyAD. The shared environmental component of anyAD was 0.09 (95% CI = 0.05–0.13). The genetic correlation of ADHD and anyAD was 0.13 (95% CI = 0.09–0.17), whereas the unique environmental correlation was almost zero at 0.02 (95% CI=-0.03–0.06). Genetic correlation explained most, in not all, the covariance between ADHD and anyAD, 0.92 (95% CI = 0.68–1.17).

**Table 3.** Results of quantitative genetic modelling. Estimates (95% confidence intervals) of genetic and environmental contribution to ADHD and a diagnosis of any of the investigated autoimmune diseases, and their correlations based on a model without C in ADHD (the model found most well-fitting and parsimonious)

	A	C	E
Explained variance			
ADHD	0.84 (0.83–0.86)	NA	0.16 (0.15–0.18)
Any of the autoimmune diseases	0.23 (0.15–0.32)	0.09 (0.05–0.13)	0.68 (0.64–0.72)
Correlation			
ADHD and any of the autoimmune diseases	0.13 (0.09–0.17)	NA	0.02 (−0.03–0.06)
Explained correlation			
ADHD and any of the autoimmune diseases	0.92 (0.68–1.17)	NA	0.08 (−0.16–0.32)

As the shared environment component of ADHD has been set to 0 in the model specification, there is no shared environment correlation or proportion of covariance to calculate.

A, additive genetic; C, shared environment; E, unique environment; NA, not applicable.

## Discussion

Our comprehensive population-based multigenerational family study found ADHD to be associated with several ADs, both within individuals and across biological relatives. ADHD was to some degree more strongly associated with maternal than paternal ADs, but by utsing aunts and uncles in a genetically informative study design, we demonstrate that this difference cannot be readily explained by AD-mediated maternal effects. Quantitative genetic modelling further indicates that the familial co-aggregation of ADHD and ADs is partly due to shared genetic factors.

In light of our results, shared genetic liabilities between ADHD and ADs seem plausible. As the heritability of ADHD is 70–80% and the role of shared environmental factors in ADHD’s aetiology has been demonstrated to be limited,^3^^,^^4^ environmental effects confounding the estimates profoundly seem improbable. Important residual environmental confounding is made further unlikely with the moderately high ORs in the primary analyses.^39^ In addition, biological aunts, uncles and cousins must be assumed to share little environment with the index individuals, in further support of shared genetic factors underlying the familial co-aggregation. Moreover, both epidemiological and molecular genetics studies have demonstrated positive genetic correlations between ADHD and ADs, in agreement with our findings.^2^^,^^13^^,^^22^ Nonetheless, we cannot rule out a role of environment and its interplay with genetic factors in explaining the observed pattern of familial co-aggregation, as genetically closer individuals tend to share more environmental factors as well. The sensitivity analyses where we adjusted for the relatives’ ADHD and the index individuals’ anyAD with little changes to the ORs, in addition to the finding of little to no individually unique environmental correlation between ADHD and anyAD in the quantitative genetic modelling, indicate that any shared aetiological factors do not operate primarily through ADHD-related behaviours or liability to ADs (e.g. early life immune dysfunction).^40–46^

However, the sensitivity analyses where we adjusted for ADHD in the relatives would not adjust for all ADHD liability/symptoms in the study population, and we cannot rule out that behaviours secondary to ADHD symptoms, e.g. smoking, again increase the risk of AD, and as such partly explain the noted familial co-aggregations. This is an area where more research is needed. It could also be that pronounced ADHD symptoms of an individual might lead to more familial stress that, in turn, increases the risk of ADs among the closest relatives, especially in mothers who are the primary caregivers. Still, it seems unlikely that this could explain all of the familial co-aggregation between ADHD and ADs, as we note familial co-aggregation in relatives as distant as full cousins. Several neuropsychiatric disorders have been associated with both ADHD^4^ and ADs,^46^^,^^47^ and thus our findings could be due to a common aetiological factor of neuropsychiatric disorders and ADs, and not represent patterns unique to ADHD. However, this is explored in the sensitivity analysis, where the remaining signs of familial co-aggregation between ADHD and ADs after the removal of all index individuals diagnosed with a non-ADHD psychiatric disorder, suggest the presence of an ADHD-specific factor (see Supplementary information, available as Supplementary data at *IJE* online). Of note, a large number of individuals were excluded from these analyses; for example, from the index cohort 70.5% of those diagnosed with ADHD were removed, with consequential bias and reduced statistical power.

The association between ADHD and maternal anyAD was stronger than the association between ADHD and paternal anyAD (and similar findings were noted for four specific ADs). Therefore, one could argue that our results support a role for AD-mediated maternal effects in the aetiology of offspring ADHD, in line with experimental studies in rodents and non-human primates that suggest that gestational maternal immune activation (MIA) may cause *in utero* neurodevelopmental disturbances in the offspring.^26^ Still, caution must be exercised in the interpretation of our findings in favour of maternal effects, including MIA. It could be that the stronger association between ADHD and maternal AD, relative to the association between ADHD and paternal AD, are due to unknown biases and/or sex effects, as well as complex genetic effects. In support of sex-dependent factors and/or biases, the association between ADHD and aunt anyAD was elevated as compared with the association between ADHD and uncle anyAD (and two specific ADs), and because this analysis was not conditioned on parental side, we suspect unknown sex effects/biases to underlie the stronger association between ADHD and aunt anyAD. Furthermore, if maternal ADs have a causal role in offspring ADHD, the maternal relatives of those with ADHD should have more ADs than the paternal relatives, as the ADs themselves have high heritabilities.^13^^,^^40^^,^^48^ Yet, in the analyses where we compared the association between ADHD and maternal aunt and uncle anyAD with the association between ADHD and paternal aunt and uncle anyAD, we did not find stronger association with the maternal side relatives’ anyAD. Nonetheless, our findings do not exclude the possibility of AD-mediated maternal (including *in utero*) effects to have a role in ADHD, but merely demonstrate that other factors should be taken into consideration before attributing maternal-paternal discrepancies in ORs, hazard ratios or risk ratios to maternal AD-mediated environment. In further opposition to a causal role of MIA in ADHD, a sibling-controlled study could not find maternal infections during pregnancy to be associated with offspring ADHD.^49^ However, similar sibling-controlled studies on AD-mediated maternal effects and ADHD are likely unsuited (including with the data available to us), as date of AD diagnosis is not a good proxy for time of AD debut, as ADs may have long prodromal phases^50^^,^^51^ in addition to which diagnostic delays are common.^52^^,^^53^ Future studies that seek to illuminate whether ADs are causal maternal risk factors for offspring ADHD and other psychiatric disorders, should use study designs that can better handle familial liability, such as those based on assisted reproduction technology.^54^

Despite the fact that both the within-individual and the index-relative ADHD-AD associations were not miniscule on the OR scale, they were still substantially smaller than what is typically reported between ADHD and other neuropsychiatric disorders.^4^^,^^5^^,^^55^ Therefore, with the low prevalences of ADs in the general population, the ARs remained small. For example, individuals with ADHD had an approximately 40% increased risk of developing celiac disease and the calculated AR of having celiac disease in our birth cohort was 1.0% among individuals with ADHD compared with 0.7% among those without ADHD, equivalent to an RD of 0.3% points. Based on our findings, with the low ARs and RDs and limited expected benefit, screening for ADs in asymptomatic individuals diagnosed with ADHD (or *vice versa*) and their relatives is not warranted.

Some of this study’s findings are replications of previous findings in independent materials.^12^^,^^17^^,^^18^^,^^20^ Still, the magnitude of the associations differs between the studies, and some previous findings were not replicated. Such variation could be due to unique characteristics of the different study populations (e.g. data sources, age, ethnicity, culture) and modelling techniques (e.g. which covariates to adjust for and how). Moreover, recent Swedish studies with similar research aims have used many of the same registries as in our study but have focused exclusively on the associations between ADHD and first-degree relatives’ AD,^21^ or only within individuals and siblings.^16^^,^^19^ We extend on these previous studies by presenting estimates for both within-individual associations and familial associations for first-, second- and third-degree relatives for many ADs, and we provide new insights as to why associations between ADHD and maternal ADs may be stronger than associations between ADHD and paternal ADs, and we present methodology that can be employed by researchers with similar data who wish to pursue similar questions. Furthermore, we present estimates of ARs and RDs that are of clinical interest, and we demonstrate extensive sensitivity analyses that indicate our findings cannot be easily explained away by such as bias or misdiagnosis. In addition, we performed quantitative genetic modelling on the familial co-aggregation between ADHD and anyAD.

Studies employing alternative study designs, e.g. insurance data and molecular genetics, have reported findings that are in direct contrast to our study.^2^^,^^13^^,^^22^ Nevertheless, such studies could be hampered by several methodological issues that may cause misleading estimates, e.g. collider bias, that is bias due to conditioning on a common outcome.^56^ Individuals with chronic health conditions, such as ADHD and ADs, may be prevented from obtaining private health insurance, and thus such data may be inherently conditioned towards individuals with a lower burden of chronic diseases, which could cause collider bias leading to negative associations/correlations between ADHD and ADs.^13^ Likewise in the ADHD GWAS, somatic comorbidity was an exclusion criterion in at least one of the sub-samples.^2^ Such criteria might introduce collider bias in studies that use summary statistics from GWASs, for example polygenic scores and whole-genome genetic correlations.^2^^,^^22^ As we used population-wide registers from a publicly funded health care system, many potential sources of bias should be minimized.

To avoid bias we excluded individuals who died or emigrated before age 10, as ADs seldom debut earlier than this age and those that do are often followed up in specialist health care and are thus recorded in the registers. Setting the cut-off age higher could lead to bias since ADHD is associated with increased mortality.^57^ As a definition of ADHD similar to ours is highly correlated with ADHD symptoms, our ADHD definition can be considered validated^55^ and many of the ADs have been validated.^31^^,^^58–61^ Moreover, we used information on prescribed medication to improve the positive predictive values of some of the AD definitions where possible. The limitation to ADs with more than 2000 cases in the birth cohort should also provide some protection from incorrectly recorded diagnoses and spurious associations.^31^^,^^62^ Sensitivity analyses with more restrictive AD definitions yielded, with few exceptions, results almost identical to the main analyses. Therefore, erroneous and tentative diagnoses probably had a limited impact on the observed findings. Moreover, the sensitivity analyses investigating geographical factors, period effects and right-censoring did not uncover worrying results, indicating that these factors did not introduce substantial bias to our study.

To conclude, we demonstrate within-individual and familial co-aggregation of ADHD and several ADs, likely reflecting shared genetic factors. Moreover, we demonstrate contrasting findings of of AD-mediated maternal effects, including MIA, having a causal role in the development of offspring ADHD. Our results have implications for the understanding of ADHD’s aetiology, but do not warrant screening for ADs among asymptomatic individuals with ADHD.

## Data availability

The population-wide individual-specific register data underlying this article were provided by Socialstyrelsen and may not be shared with outside researchers. Data may be obtained after application to Socialstyrelsen [https://www.socialstyrelsen.se/en/].

## Supplementary data

Supplementary data are available at *IJE* online.

## Funding

This study has received funding from: Stiftelsen Kristian Gerhard Jebsen (K.K., J.H); the University of Bergen (T.A.H., K.K., J.H.); the Western Norway Regional Health Authorities (J.H.); the Swedish Research Council Starting Grant (A.B., grant number 2017–00788); Fredrik och Ingrid Thurings Stiftelse (A.B., grant number 2016–00254); the Swedish Initiative for Research on Microdata in the Social And Medical Sciences (SIMSAM) framework (C.A., H.L., grant number 340–2013-5867); the Strategic Research Programme in Neuroscience (StratNeuro) of Karolinska Institutet (A.B.); the Swedish Research Council (H.L, grant number 2014–3831); the Swedish Brain Foundation (H.L., grant number FO2018-0273); and the European Union’s Horizon 2020 research and innovation programme [K.K., S.V.F., J.H., H.L., grant agreement number 667302 (CoCA)]. None of the funding sources had any involvement in any parts of this study.

## Conflict of interest

J.H. has served as a speaker for Eli-Lilly, Shire, HB Pharma, Medice and Biocodex. H.L. has served as a speaker for Eli-Lilly and Shire and has received research grants from Shire, all outside the submitted work. None of the other authors have any conflicts of interests to declare.

## Supplementary Material

dyab151_Supplementary_Data
