# Supplementary material for: Familial co-aggregation of attention-deficit/hyperactivity disorder and autoimmune diseases: a cohort study based on Swedish population-wide registers
Source: Int J Epidemiol. 2021 Aug 11;51(3):898–909. doi: 10.1093/ije/dyab151 (PMC9189956; doi:10.1093/ije/dyab151)
Supplement: dyab151_Supplementary_Data [file dyab151_supplementary_data.pdf]

Supplementary information

Familial co-aggregation of attention-deficit/hyperactivity disorder and autoimmune diseases: a cohort study based on Swedish population-wide registers

Table of Contents

Rationales and descriptions of sensitivity analyses: 2

Supplementary Table S1: Case definitions for disorders/diseases in the main analyses. 4

Supplementary Table S2: Case definitions for disorders/diseases in the sensitivity analyses. 6

Supplementary Table S3: Sex-specific within individual associations between attention-deficit/hyperactivity disorder (ADHD) and autoimmune diseases. 7

Supplementary Figure S1: The prevalences of the diseases and disorders investigated per birth year of the index individuals by sex. 8

Supplementary Figure S2: All associations between index individuals' attention-deficit/hyperactivity disorder (ADHD) and index or relatives' autoimmune disease as odds ratios with 95% confidence intervals and P-values (adjusted for year of birth of index and relative if applicable). 9

Supplementary Figure S3: The associations between index individual attention-deficit/hyperactivity disorder (ADHD) and index or relatives' autoimmune disease calculated as standardized absolute risks and standardized risk differences with 95% confidence intervals (adjusted for year of birth of index and relative if applicable) 10

Supplementary Figure S4: The interaction by parent type on the association between index attention-deficit/hyperactivity disorder (ADHD) and autoimmune disease as exponentiated coefficients with 95% confidence intervals and P-values (adjusted for year of birth of index and relative). 13

Supplementary Figure S5: The associations between index individual attention-deficit/hyperactivity disorder (ADHD) and relatives' autoimmune disease with adjustment for relatives' ADHD (and year of birth of index and relative if applicable). 14

Supplementary Figure S6: The associations between index individual attention-deficit/hyperactivity disorder (ADHD) and index or relatives' autoimmune disease after exclusion of all index individuals who have received any non-ADHD psychiatric disorder diagnosis in specialist health care (adjusted for year of birth of index and relative if applicable). 15

Supplementary Figure S7: The associations between index individual attention-deficit/hyperactivity disorder (ADHD) and index or relatives' autoimmune disease (adjusted for year of birth of index and relative if applicable). The case definition for all autoimmune diseases required at least two recordings in the Swedish national patient register. 16

Supplementary Figure S8: The associations between index individual attention-deficit/hyperactivity disorder (ADHD) and index or relatives' celiac disease, Crohn's disease and ulcerative colitis after exclusion of individuals who have received a diagnosis of irritable bowel syndrome (adjusted for year of birth of index and relative if applicable). 17

Supplementary Figure S9: The associations between index individual attention-deficit/hyperactivity disorder (ADHD) and index or relatives' autoimmune disease with adjustment for the county of birth of the index individuals (and for year of birth of index and relative if applicable). 18

Supplementary Figure S10: The associations between index individual attention-deficit/hyperactivity disorder (ADHD) and index or relatives' autoimmune disease (adjusted for year of birth of index and relative if applicable). The case definitions of the autoimmune diseases only included Swedish national patient register recordings from 2001 or later. 19

Supplementary Figure S11: The associations between index individual attention-deficit/hyperactivity disorder (ADHD) and index or relatives' autoimmune disease (adjusted for year of birth of index and relative if applicable) after restricting to index individuals born 1987-2010 20

References 21

Rationales and descriptions of sensitivity analyses:

It should be noted that both within individual and familial co-aggregation of attention-deficit/hyperactivity (ADHD) and autoimmune diseases (ADs), could be a measure of the direct effects of ADHD on AD. E.g., individuals with ADHD are known to smoke more tobacco[1, 2] and weigh more[3], both of which have been hypothesized to be causal in the development of several ADs[4-6]. Hence, familial co-aggregation of ADHD and ADs could be due to a combination of familial aggregation of ADHD[7, 8] and the direct effects of relatives' ADHD on the same relatives' ADs. To test the existence of common underlying familial liability to ADHD and ADs, we repeated the family analyses with adjustment for the relatives' ADHD as a covariate. The resulting estimates are biased, but no substantial attenuation of the estimates (ORs) and remaining statistical significance would support the hypothesis of a shared familial liability between ADHD and AD. Detailed rationales supporting these analyses have previously been published[9, 10]. After adjustment for relatives' ADHD, there were only subtle changes in the familial association results. See Supplementary Figure S5. Likewise there could be a direct effect of autoimmunity on ADHD. We therefore repeated the family analyses with adjustment for any of the autoimmune diseases (anyAD) in the indexes. This adjustment did not have a profound impact on the familial associations. The results are not presented.

Besides attention-deficit/hyperactivity (ADHD), several other psychiatric disorders have been associated with ADs [11-15]. To assess whether ADHD was independently associated with ADs, we repeated the within individual and family analyses after the exclusion of all index individuals who had ever been diagnosed with a psychiatric disorder. See Supplementary Table S2 for case definition. In these analyses many of the associations were attenuated and the 95%CIs were much wider. However, several within individual and index-relative ADHD-AD associations remained statistically significant. See Supplementary Figure S6.

ADHD medication has been used off-label to treat multiple sclerosis symptoms. If ADHD medication has been used off-label in the treatment of other ADs, this may lead to false positive associations due to our definition of ADHD case status. To confirm this was not the case, we repeated the within individual and family analyses with ADHD defined only from the Swedish National Patient Register. In these analyses, multiple sclerosis was defined from the Swedish National Patient Register solely without the exclusion of individuals who had dispensed ADHD medication. These sensitivity analyses did not have a profound impact on any associations. The results are not presented.

Some diagnoses of the respective ADs could be the results of tentative diagnostics, and ADHD patients may be more likely to get tentative diagnoses as they might be in more contact with health services, potentially leading to false positive associations. To reduce the occurrence of tentative diagnoses, we performed sensitivity analyses where the AD case definitions required at least two recordings of the specific ADs. Individuals with only one recording of an AD were defined as not having that specific AD. After requiring two diagnoses, there was little to no remaining signal for any association between ADHD and ulcerative colitis, neither within individuals nor across relatives, with ORs clustering around 1.0 and no P-values lower than 0.1. Regarding the remaining ADs, most of the within individual associations were somewhat attenuated with the requirement of two diagnoses, though the associations between index ADHD and relative's ADs were robust. See Supplementary Figure S7.

ADHD patients are perhaps more likely to have psychosomatic illnesses, and symptoms of psychosomatic disorders may mimic ADs, which could lead to false positive associations between ADHD and ADs. Therefore, we repeated the within individual analyses with the exclusion of individuals who had received a diagnosis of psychosomatoform disorder, and the family analyses with the exclusion of relatives who had received a psychosomatoform disorder diagnosis. See Supplementary Table S2 for case definition. These sensitivity analyses did not have a profound impact on any associations. The results are not presented.

Irritable bowel disease may mimic celiac disease, Crohn's disease and ulcerative colitis. For these three ADs, we therefore repeated the within individual analyses

with the exclusion of individuals who had received a diagnosis of irritable bowel syndrome, and the family analyses with the exclusion of relatives who had received a diagnosis of irritable bowel syndrome. See Supplementary Table S2 for case definition. In these analyses, ADHD's associations with ulcerative colitis were attenuated with ORs clustering around 1.0, and no P-values lower than 0.09. For celiac disease and Crohn's disease there were only minor changes to the estimates. See Supplementary Figure S8.

The risk of receiving an ADHD diagnosis may vary geographically across Sweden[16]. Likewise, the risk of receiving a diagnosis of an AD might also vary geographically[17]. Consequently, location of birth and living might confound the associations between ADHD and ADs. To confirm that our results were not driven by geographic factors, we utilized the Swedish Medical Birth Register which was established in 1973[18] to attain information on the county of birth of the index individuals. We then repeated the familial co-aggregation analyses with adjustment for county of birth of the index individuals (born 1973-2010). The results were generally in line with the main findings. See Supplementary Figure S9.

The Swedish National Patient Register did not include outpatient visits until 2001[19], and inpatient diagnoses may represent more serious disease states. Since this may lead to bias, we repeated the within individual and family analyses by using only AD recordings made in 2001 or later. After this restriction, attenuation of the estimates was noted in parent generation celiac disease, but not in the index generation. For the remaining ADs, there were only mostly minor attenuation of the ORs and wider 95%CIs. See Supplementary Figure S10. Similarly, as the Swedish National Patient Register did not register ADHD prior the introduction of the international classification of diseases 9th revision (ICD-9) in 1987, immortal time bias could potentially be introduced in the analyses. E.g. an individual born in 1960 could not have been diagnosed with ADHD before age 27. In light of this issue, we repeated the within individual and family analyses after restricting to index individuals born 1987 to 2010. The results were largely in line with the main analyses, albeit somewhat attenuated with wider confidence intervals. See Supplementary Figure S11.

Right-censoring could potentially bias our study as many individuals would be too young to have been diagnosed with ADHD or ADs. Thus, we repeated the within individual, sibling and cousin analyses with restriction to index individuals born between 1960 and 1990. These sensitivity analyses did not have a profound impact on any associations. The results are not presented.

Supplementary Table S1: Case definitions for disorders/diseases in the main analyses.

Disorder/disease	Definition
ADHD	At least one diagnosis of ICD-9: 314<br>or ICD-10: F90<br>or dispensed at least once ATC: N06BA01, N0BA02, N06BA04, N06BA09, N06BA12
Any of the 13 autoimmune diseases (anyAD)	Fulfilled the required definition of at least one the 13 specific autoimmune diseases
Ankylosing spondylitis	At least one diagnosis of ICD-8: 712.4<br>or ICD-9: 720A<br>or ICD-10: M45
Celiac disease	At least one diagnosis of ICD-8: 269.00, 269.99<br>or ICD-9: 579A<br>or ICD-10: K90.0
Crohn's disease	At least one diagnosis of ICD-8: 563.00<br>or ICD-9: 555<br>or ICD-10: K50
Grave's disease	At least one diagnosis of ICD-8: 242.00<br>or ICD-9: 242A<br>or ICD-10: E05.0
Hashimoto's disease	At least one diagnosis of ICD-8: 245.03<br>or ICD-9: 245C<br>or ICD-10: E06.3<br>If alive after 2006, also required one dispensed drug with ATC: H03AA to avoid false positives
Multiple sclerosis	At least one diagnosis of ICD-8: 340<br>or ICD-9: 340<br>or ICD-10: G35<br>In addition, all individuals who had ever dispensed an ADHD-drug, but never ICD-9: 314 or ICD-10: F90 were set to missing for this variable to avoid off-label prescribed stimulants

Psoriasis	At least one diagnosis of ICD-8: 696.10, 696.19<br>or ICD-9: 696B<br>or ICD-10: L40.0, L40.1, L40.2, L.40.3, L40.8, L40.9
Rheumatoid arthritis	At least one diagnosis of ICD-8: 712.10, 712.38, 712.39, 712.50, 712.59<br>or ICD-9: 714A, 714B, 714C<br>or ICD-10: M05, M06
Sarcoidosis	At least one diagnosis of ICD-8: 135<br>or ICD-9: 135<br>or ICD-10: D86
Sjögren's disease	At least one diagnosis of ICD-9: 710C<br>or ICD-10: M35.0
Systemic lupus erythematosus	At least one diagnosis of ICD-9: 710A<br>or ICD-10: M32
Type 1 diabetes mellitus	If dead 2006 or before: At least one ICD-10: E10 and never prescribed ATC: A10B<br>If alive after 2006: At least one ICD-10: E10 and dispensed ATC: A10A at least once and never dispensed ATC: A10B
Ulcerative colitis	At least one diagnosis of ICD-8: 563.10<br>or ICD-9: 556<br>or ICD-10: K51
Abbreviations: ADHD attention-deficit/hyperactivity disorder; ATC Anatomical Therapeutic Chemical; ICD International Classification of Diseases	

Supplementary Table S2: Case definitions for disorders/diseases in the sensitivity analyses.

Disorder/disease	Definition
Any psychiatric disorder that is not ADHD	At least one diagnosis of ICD-8: 291, 292, 295, 296, 297, 298, 299, 300, 301, 303, 304, 307, 308, 310.9, 311.9, 312.9, 313.9, 314.9, 315.9<br>or ICD-9: 291, 292, 293, 294, 295, 296, 297, 298, 299, 300, 301, 302, 303, 304,305A,305X 306, 307, 308, 309, 310, 311, 312, 313, 315, 316, 317, 318, 319<br>or ICD-10: F2, F3, F4, F5, F6, F7, F8, F10, F11, F12, F13, F14, F15, F16, F18, F19, F91, F92, F93, F94, F95, F98, F99
Irritable bowel syndrome	At least one diagnosis of ICD-8: 564.19<br>or ICD-9: 564B<br>or ICD-10: K58
Psychosomatoform disorder	At least one diagnosis of ICD-8: 305<br>or ICD-9: 306<br>or ICD-10: F45
Abbreviations: ADHD attention-deficit/hyperactivity disorder; ICD International Classification of Diseases	

Supplementary Table S3: Sex-specific within individual associations between attention-deficit/hyperactivity disorder (ADHD) and autoimmune diseases.

Disorder/disease	Within individual female OR (95%CI); P-value. Adjusted for year of birth.	Within individual male OR (95%CI); P-value. Adjusted for year of birth.	P-value for interaction by sex. Adjusted for year of birth.
Any of the autoimmune diseases	1.38 (1.32-1.44); P<0.001	1.30 (1.25-1.35); P<0.001	0.127
Ankylosing spondylitis	1.53 (1.17-1.99); P=0.002	1.15 (0.92-1.44); P=0.220	0.084
Celiac disease	1.39 (1.28-1.50); P<0.001	1.38 (1.28-1.49); P<0.001	0.543
Crohn's disease	1.40 (1.23-1.59); P<0.001	1.21 (1.07-1.36); P=0.002	0.279
Graves' disease	1.31 (1.14-1.50); P<0.001	1.47 (1.13-1.90); P=0.004	0.223
Hashimoto's thyroiditis	1.58 (1.36-1.84); P<0.001	1.98 (1.55-2.53); P<0.001	0.004
Multiple sclerosis	0.78 (0.59-1.03); P=0.083	1.05 (0.75-1.46); P=0.791	0.146
Psoriasis	1.65 (1.52-1.79); P<0.001	1.38 (1.28-1.49); P<0.001	<0.001
Rheumatoid arthritis	1.16 (0.99-1.35); P=0.065	1.50 (1.22-1.84); P<0.001	0.062
Sarcoidosis	1.41 (1.04-1.91); P=0.026	1.22 (0.96-1.54); P=0.104	0.260
Sjögren's syndrome	1.93 (1.48-2.52); P<0.001	0.88 (0.36-2.15); P=0.780	0.217
Systemic lupus erythematosus	1.08 (0.80-1.45); P=0.619	0.70 (0.31-1.58); P=0.390	0.396
Type 1 diabetes	1.35 (1.22-1.50); P<0.001	1.27 (1.18-1.37); P<0.001	0.170
Ulcerative colitis	1.18 (1.04-1.33); P=0.010	1.05 (0.95-1.17); P=0.341	0.344
Abbreviations: OR odds ratio; CI confidence interval			

Supplementary Figure S1: The prevalences of the diseases and disorders investigated per birth year of the index individuals by sex.

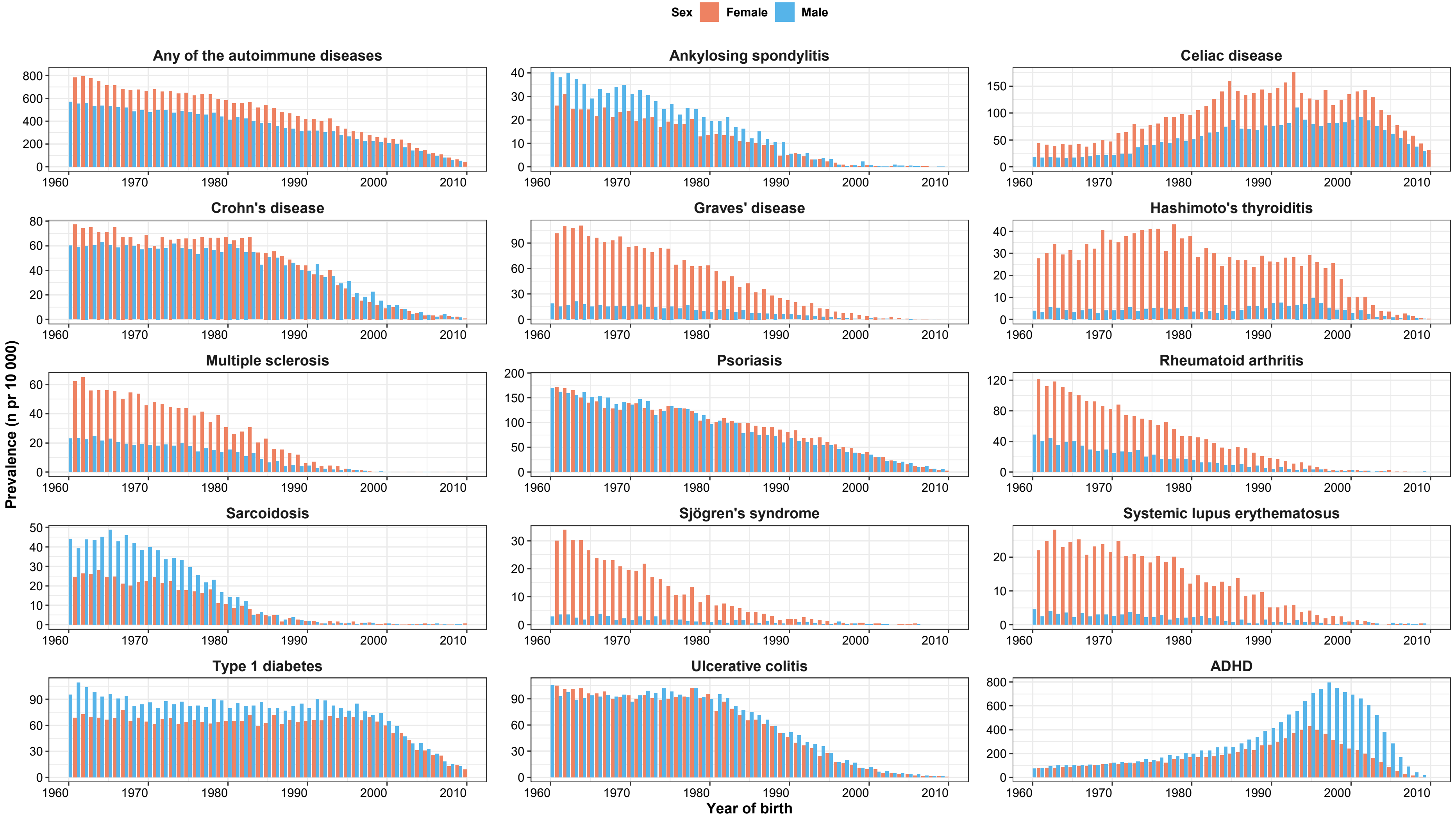

Abbreviation: ADHD attention-deficit/hyperactivity disorder

Supplementary Figure S2: All associations between index individuals' attention-deficit/hyperactivity disorder (ADHD) and index or relatives' autoimmune disease as odds ratios with 95% confidence intervals and P-values (adjusted for year of birth of index and relative if applicable). The results presented in Figure 1 are also present in this figure for comprehensiveness. The x-axes are log-transformed.

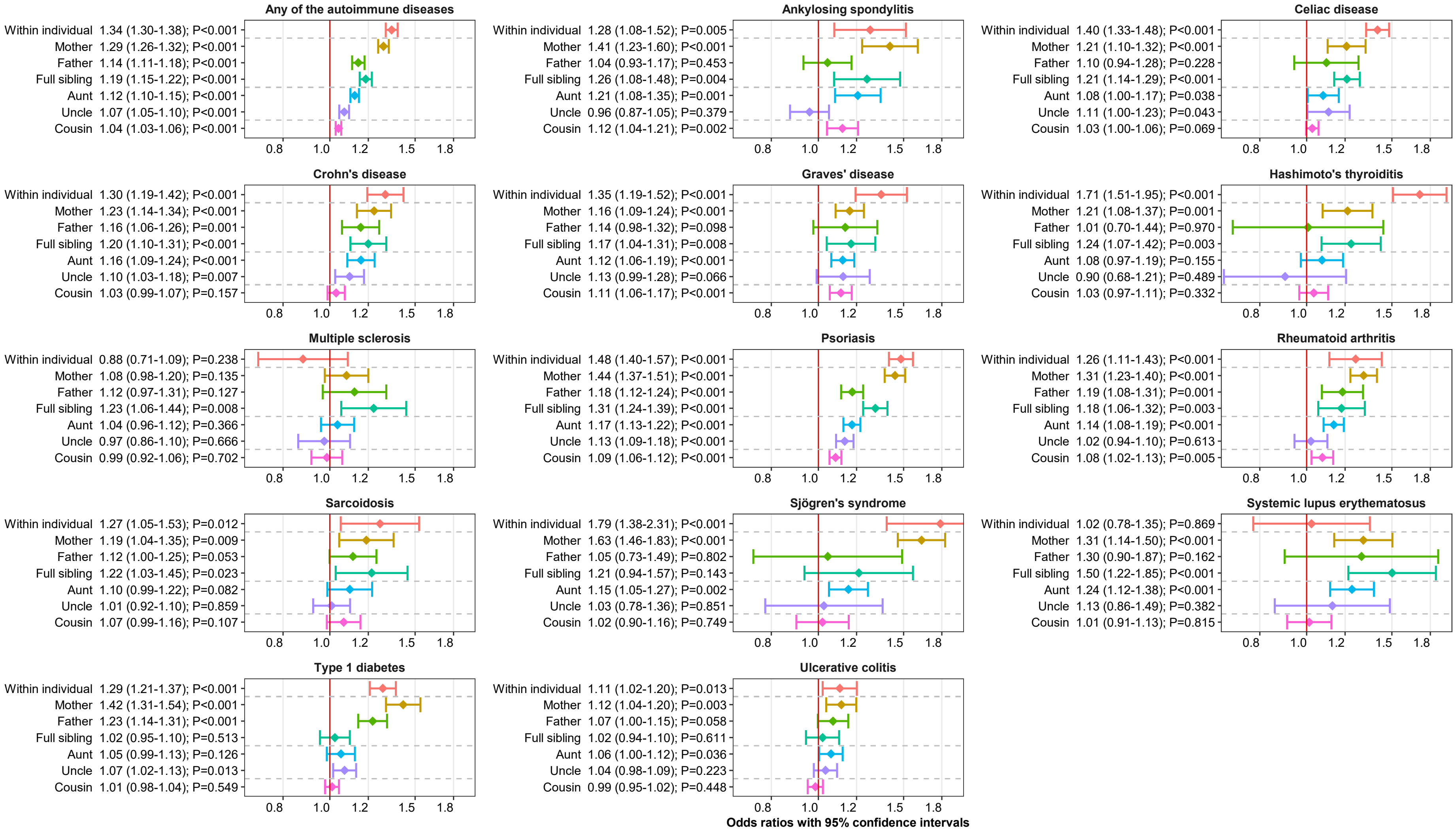

Supplementary Figure S3: The associations between index individual attention-deficit/hyperactivity disorder (ADHD) and index or relatives' autoimmune disease calculated as standardized absolute risks and standardized risk differences with 95% confidence intervals (adjusted for year of birth of index and relative if applicable). The estimates for the relatives should be interpreted as the calculated risk of having a relative with the autoimmune disease of interest, given the index individual having ADHD or not. For example, if one does not have ADHD, the absolute risk of having a mother with psoriasis is 1.57% as compared to those with ADHD, where the absolute risk of having a mother with psoriasis is 2.24%.

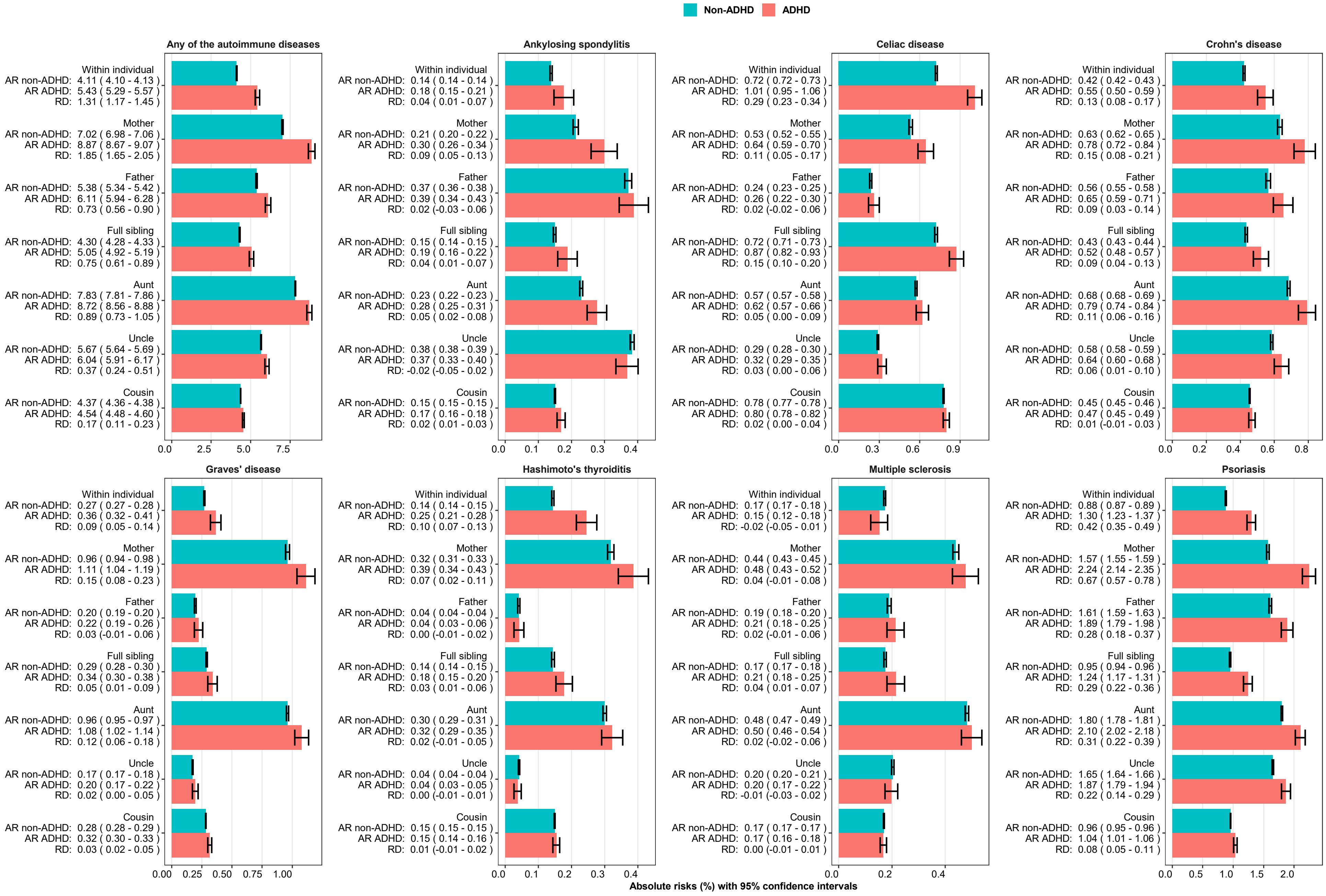
Non-ADHD
ADHD
Any of the autoimmune diseases
Ankylosing spondylitis
Celiac disease
Crohn's disease
Graves' disease
Hashimoto's thyroiditis
Multiple sclerosis
Psoriasis
Within individual
Mother
Father
Full sibling
Aunt
Uncle
Cousin
Absolute risks (%) with 95% confidence intervals

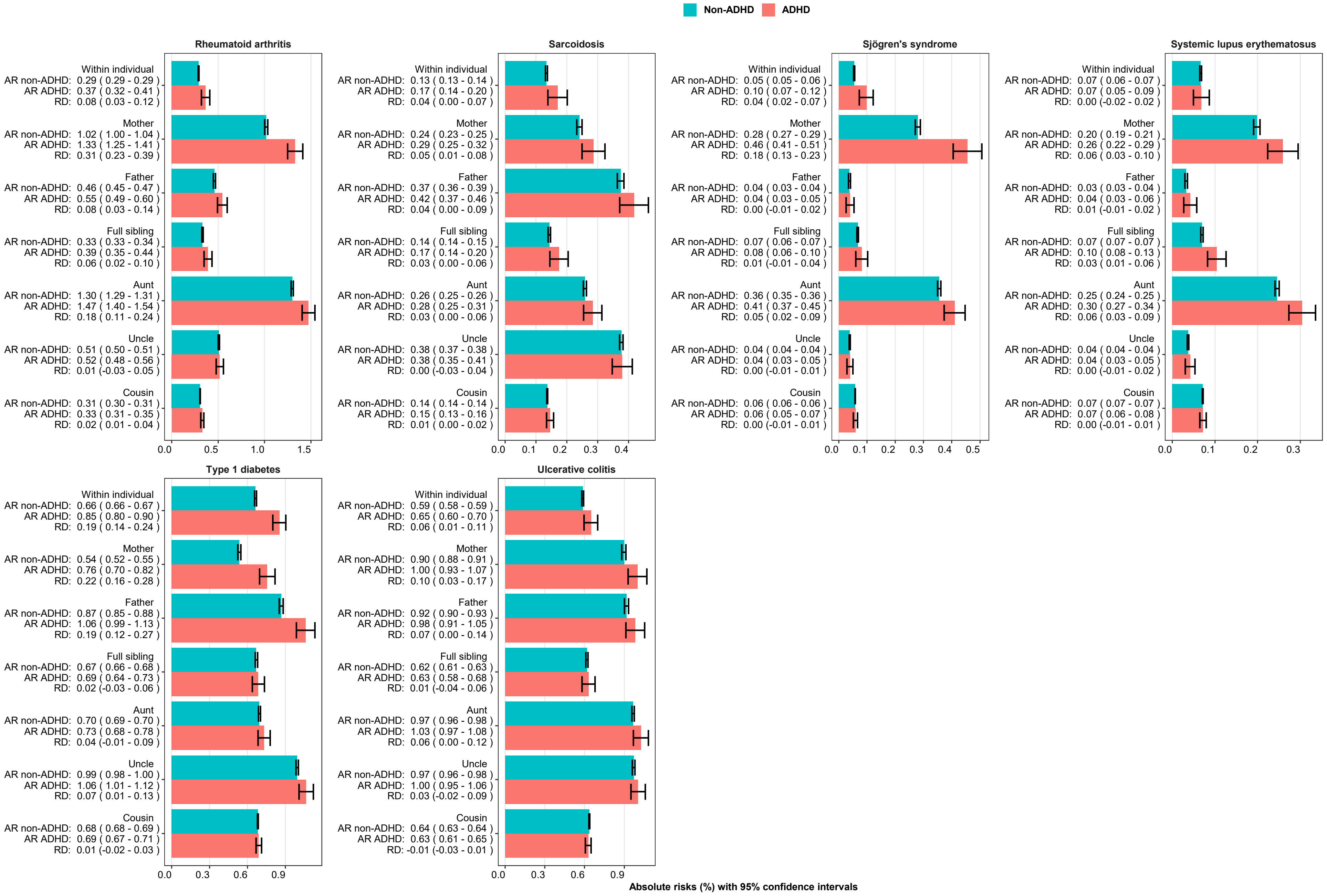

Abbreviations: ADHD attention-deficit/hyperactivity disorder, AR absolute risk, RD risk difference.

Supplementary Figure S4: The interaction by parent type on the association between index attention-deficit/hyperactivity disorder (ADHD) and autoimmune disease as exponentiated coefficients with 95% confidence intervals and P-values (adjusted for year of birth of index and relative). The exponentiated coefficient represent the ratio of the two odds ratios (OR), e.g. $OR_{index\text{-}mother}$ and $OR_{index\text{-}father}$ in the anlyses of "mother vs father". If the ratio is > 1, it indicates that $OR_{index\text{-}mother}$ is higher than $OR_{index\text{-}father}$. Similar for "aunt vs uncle", and "maternal aunt and uncle vs paternal aunt and uncle" ("maternal side vs paternal side"). The results presented in Figure 2 are also present in this figure for comprehensiveness. The x-axes are log-transformed.

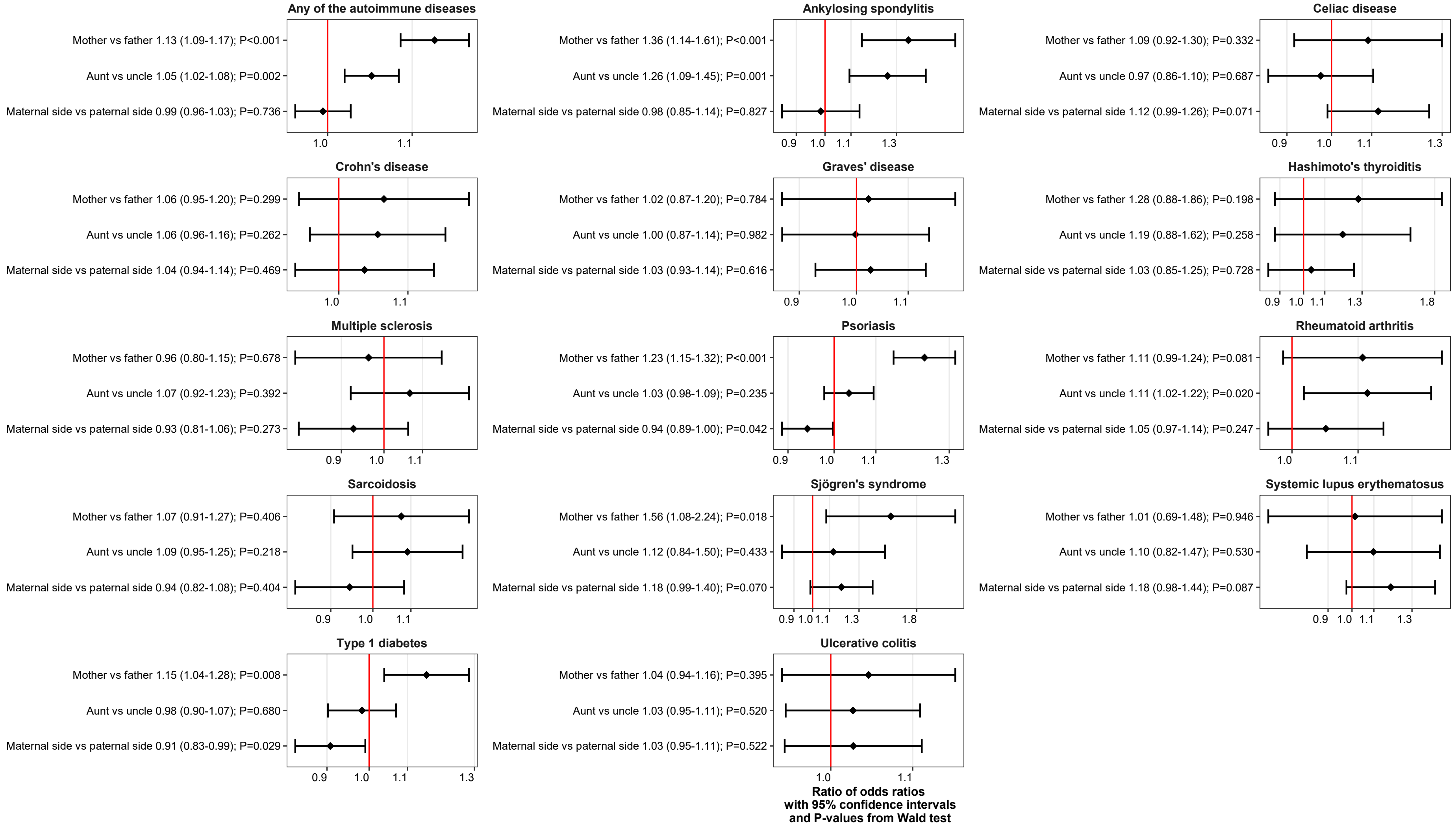

Supplementary Figure S5: The associations between index individual attention-deficit/hyperactivity disorder (ADHD) and relatives' autoimmune disease with adjustment for relatives' ADHD (and year of birth of index and relative if applicable). The intraindividual associations, which are not affected in these analyses, are included for reference. The x-axes are log-transformed.

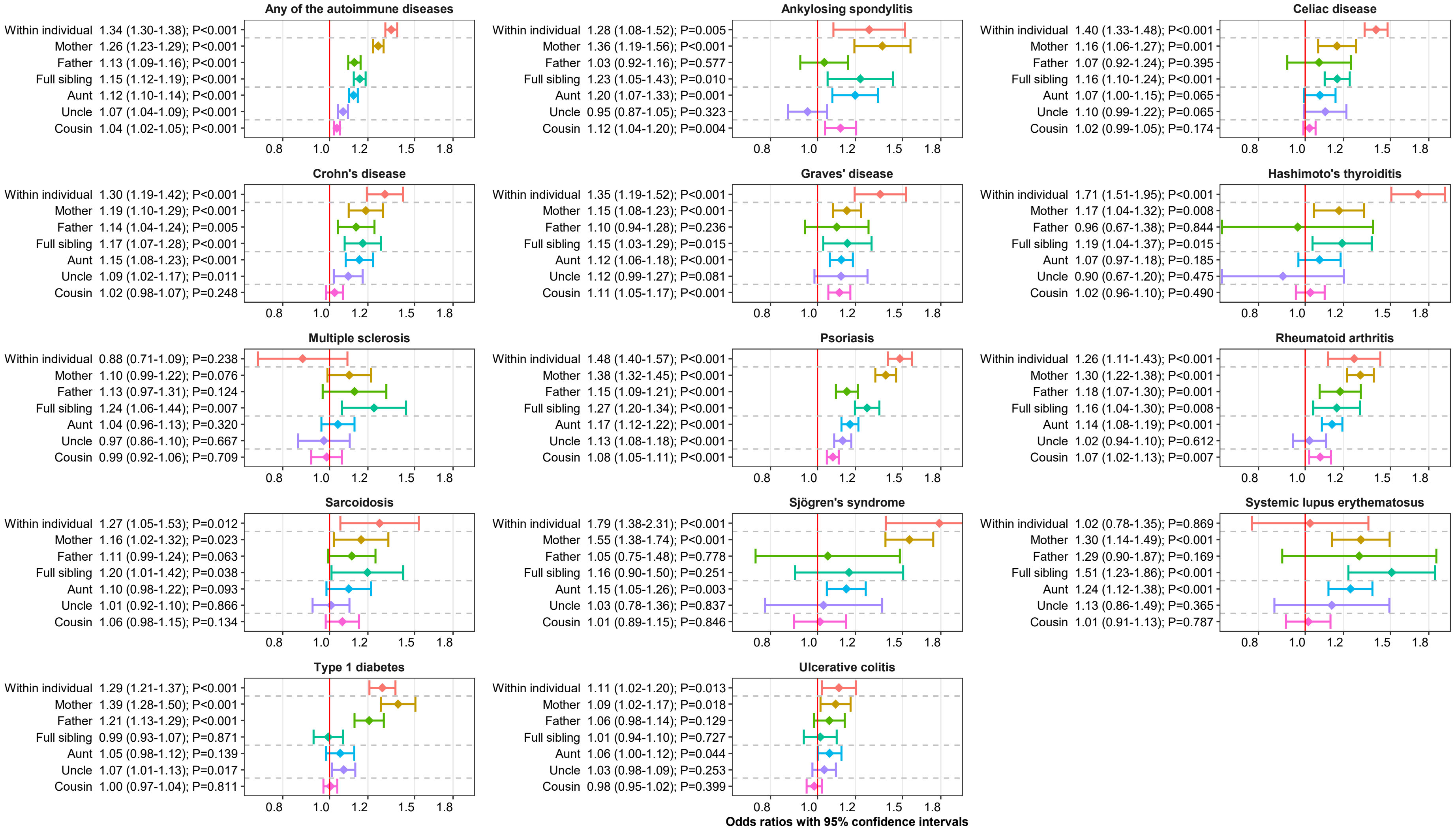

Supplementary Figure S6: The associations between index individual attention-deficit/hyperactivity disorder (ADHD) and index or relatives' autoimmune disease after exclusion of all index individuals who have received any non-ADHD psychiatric disorder diagnosis in specialist health care (adjusted for year of birth of index and relative if applicable). The x-axes are log-transformed

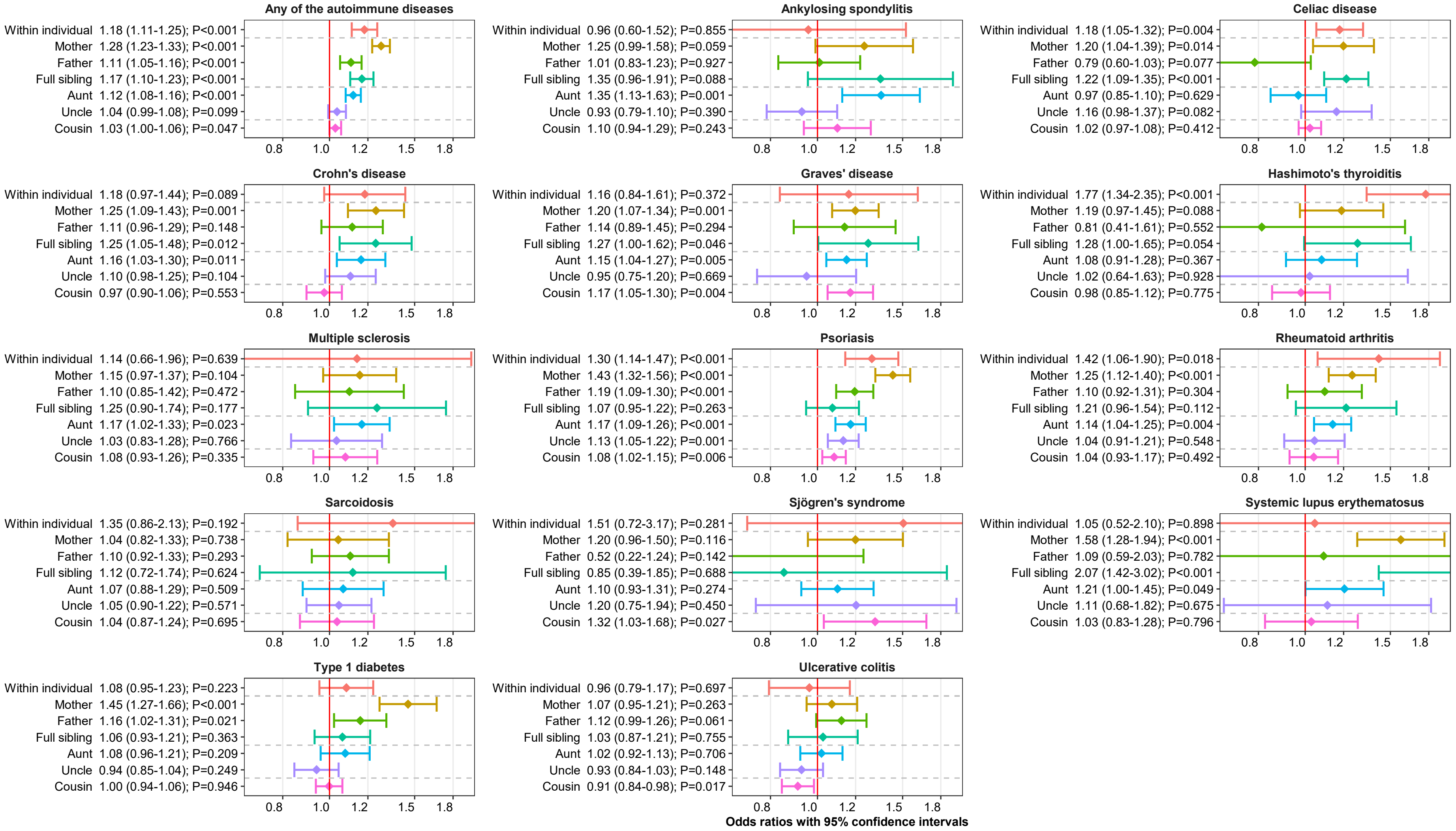

Supplementary Figure S7: The associations between index individual attention-deficit/hyperactivity disorder (ADHD) and index or relatives' autoimmune disease (adjusted for year of birth of index and relative if applicable). The case definition for all autoimmune diseases required at least two recordings in the Swedish national patient register. The x-axes are log-transformed.

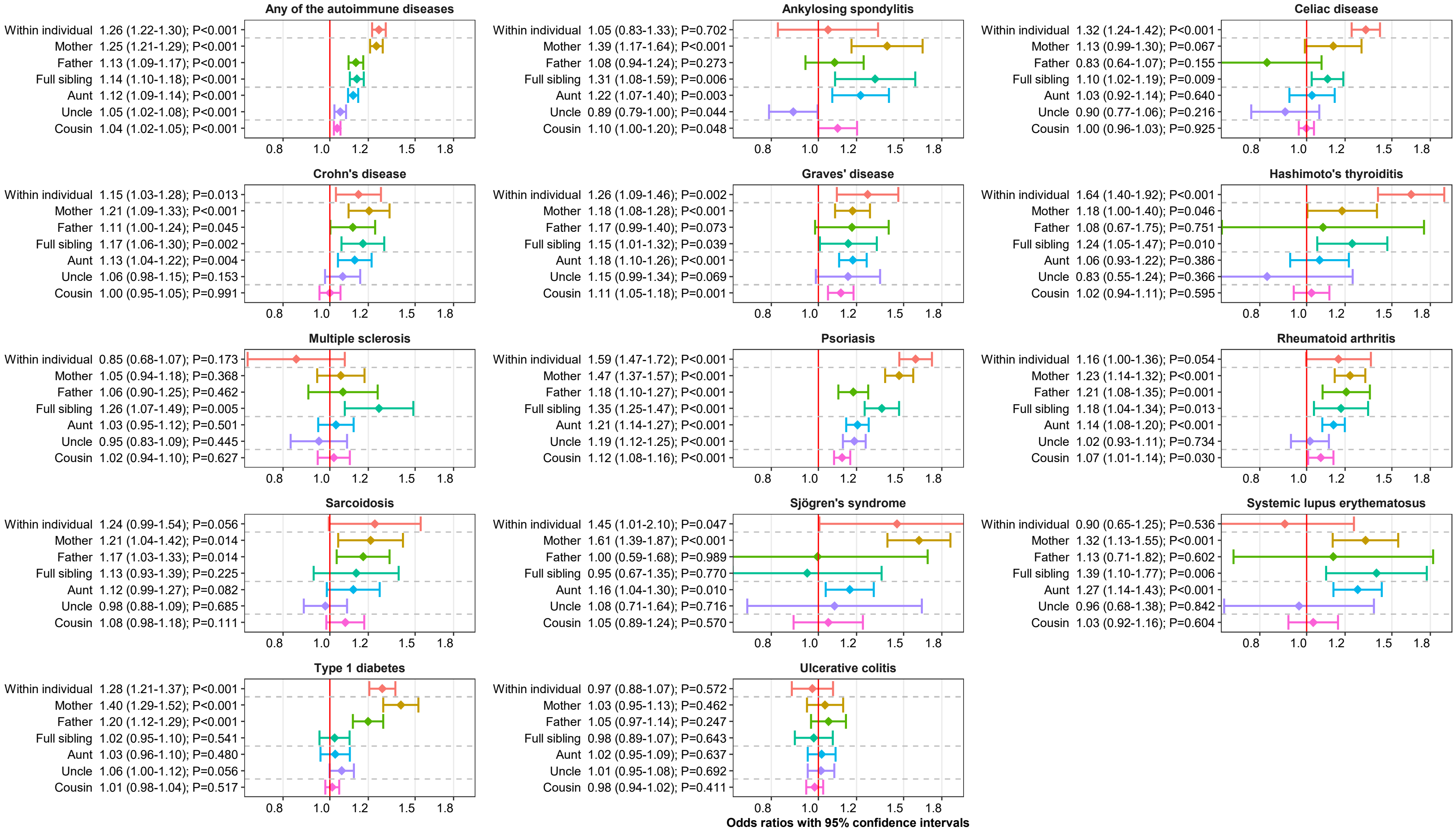

Supplementary Figure S8: The associations between index individual attention-deficit/hyperactivity disorder (ADHD) and index or relatives' celiac disease, Crohn's disease and ulcerative colitis after exclusion of individuals who have received a diagnosis of irritable bowel syndrome (adjusted for year of birth of index and relative if applicable). The x-axes are log-transformed.

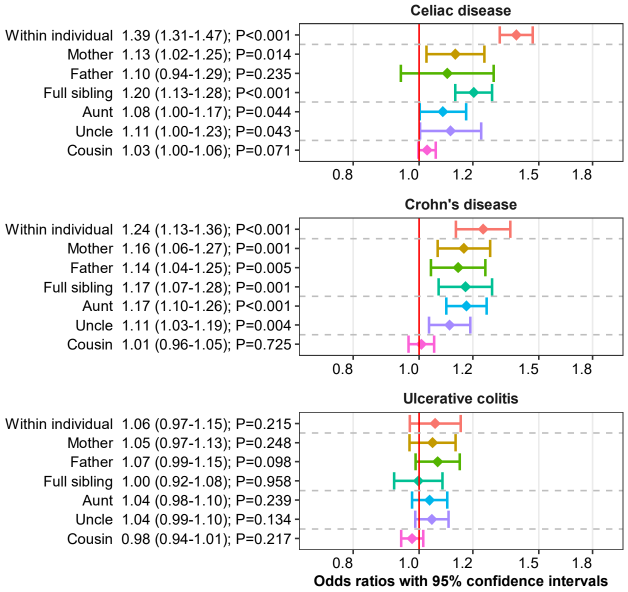

Supplementary Figure S9: The associations between index individual attention-deficit/hyperactivity disorder (ADHD) and index or relatives' autoimmune disease with adjustment for the county of birth of the index individuals (and for year of birth of index and relative if applicable). The x-axes are log-transformed.

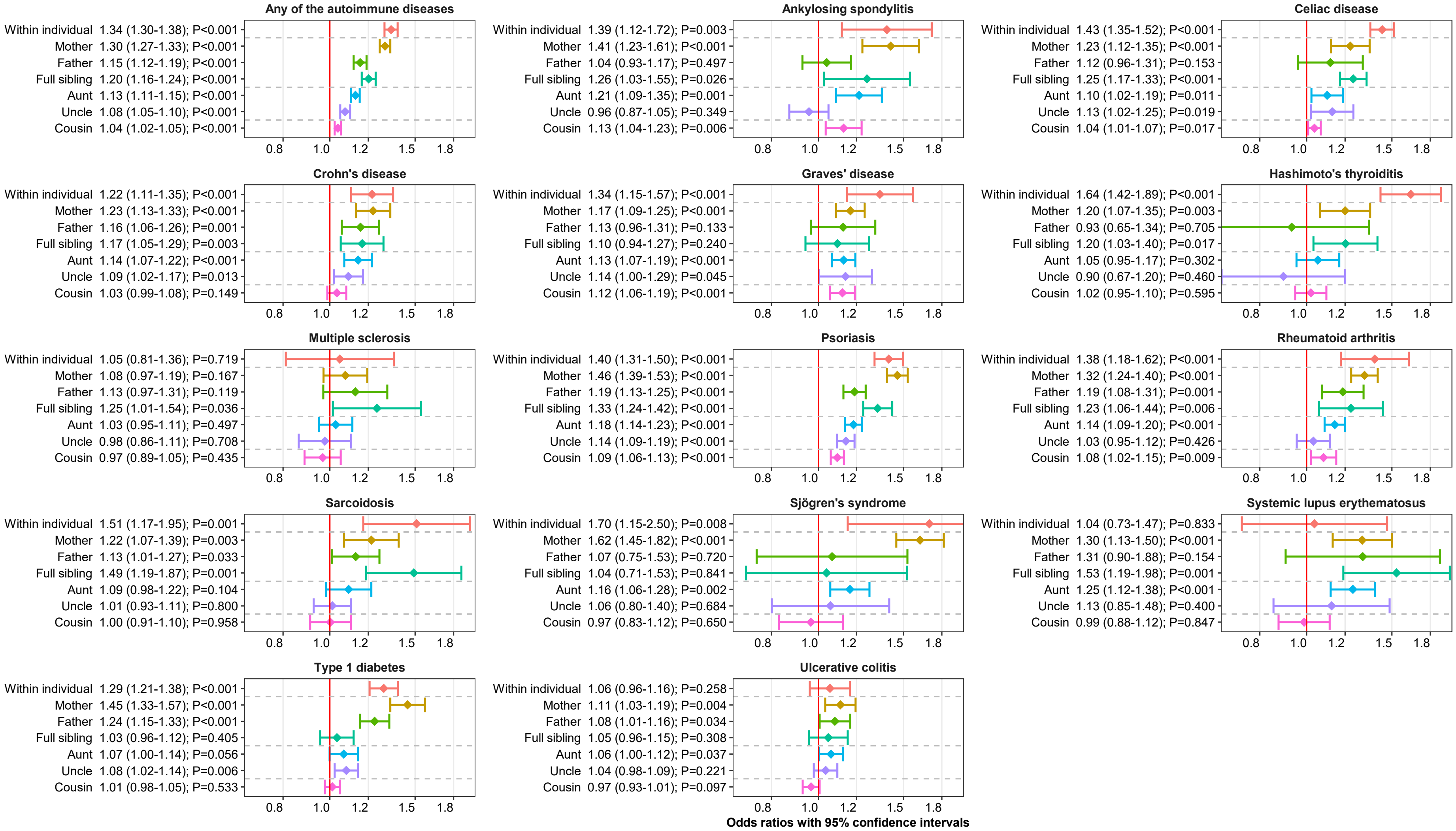

Supplementary Figure S10: The associations between index individual attention-deficit/hyperactivity disorder (ADHD) and index or relatives' autoimmune disease (adjusted for year of birth of index and relative if applicable). The case definitions of the autoimmune diseases only included Swedish national patient register recordings from 2001 or later. The x-axes are log-transformed.

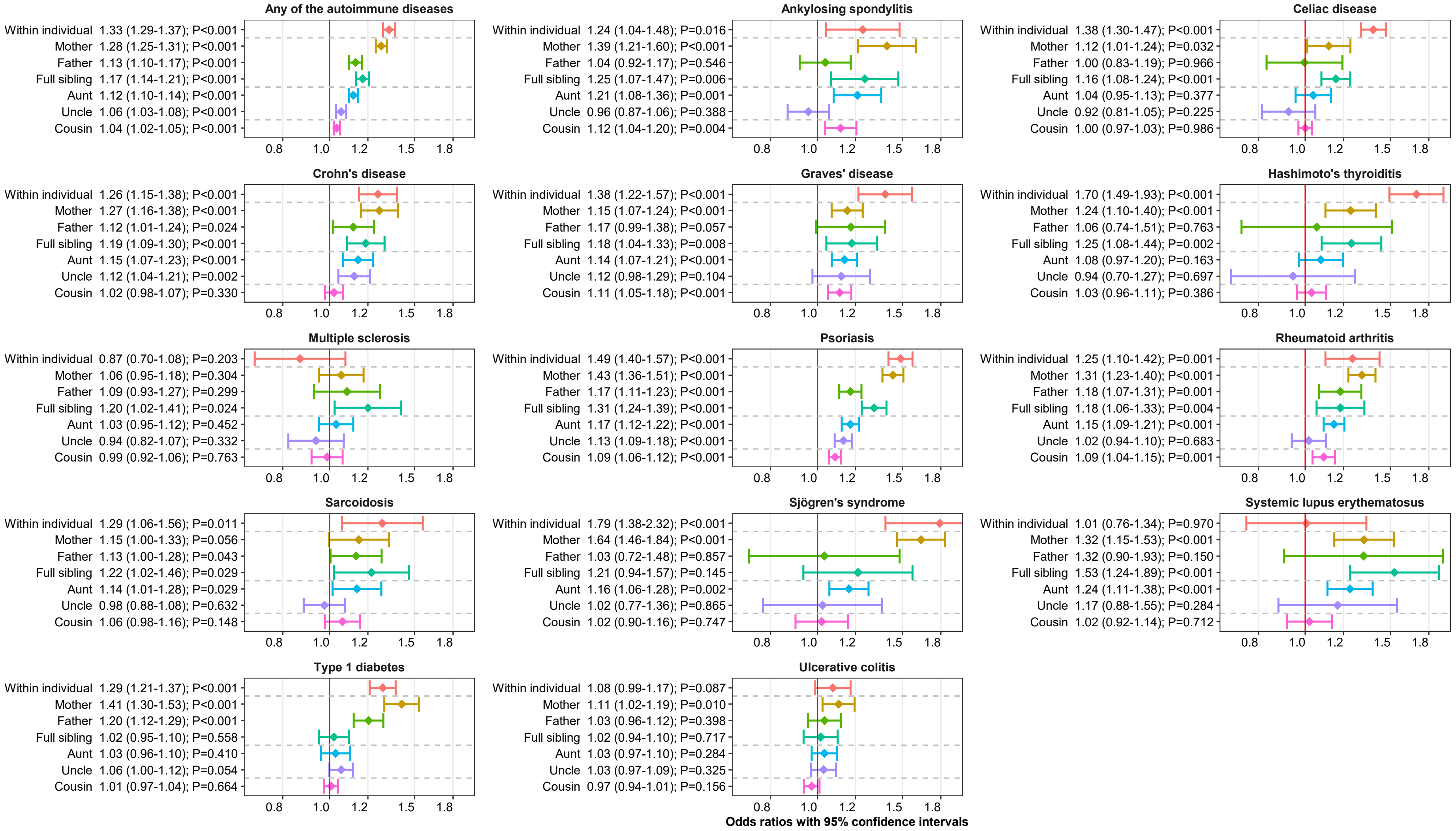

Supplementary Figure S11: The associations between index individual attention-deficit/hyperactivity disorder (ADHD) and index or relatives' autoimmune disease (adjusted for year of birth of index and relative if applicable) after restricting to index individuals born 1987-2010. The x-axes are log-transformed.

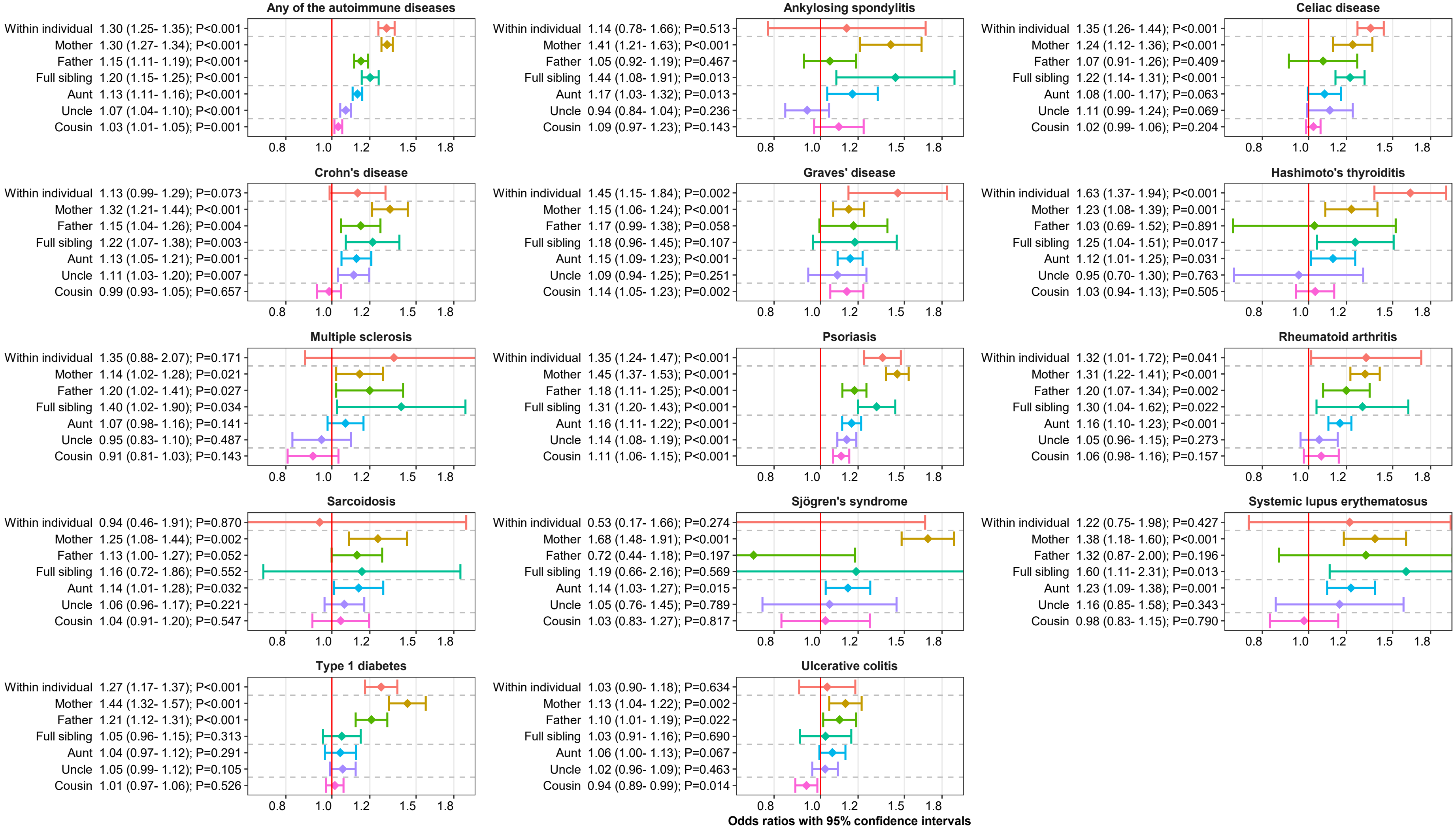

References

1. van Amsterdam J, van der Velde B, Schulte M, van den Brink W. Causal Factors of Increased Smoking in ADHD: A Systematic Review. *Subst Use Misuse* 2018; **53**: 432-45.
2. Charach A, Yeung E, Climans T, Lillie E. Childhood attention-deficit/hyperactivity disorder and future substance use disorders: comparative meta-analyses. *J Am Acad Child Adolesc Psychiatry* 2011; **50**: 9-21.
3. Chen Q, Kuja-Halkola R, Sjolander A, et al. Shared familial risk factors between attention-deficit/hyperactivity disorder and overweight/obesity - a population-based familial coaggregation study in Sweden. *J Child Psychol Psychiatry* 2017; **58**: 711-8.
4. Perricone C, Versini M, Ben-Ami D, et al. Smoke and autoimmunity: The fire behind the disease. *Autoimmun Rev* 2016; **15**: 354-74.
5. Versini M, Jeandel PY, Rosenthal E, Shoenfeld Y. Obesity in autoimmune diseases: not a passive bystander. *Autoimmun Rev* 2014; **13**: 981-1000.
6. Ananthakrishnan AN. Epidemiology and risk factors for IBD. *Nat Rev Gastroenterol Hepatol* 2015; **12**: 205-17.
7. Chen Q, Brikell I, Lichtenstein P, et al. Familial aggregation of attention-deficit/hyperactivity disorder. *J Child Psychol Psychiatry* 2017; **58**: 231-9.
8. Jokiranta-Olkoniemi E, Cheslack-Postava K, Joelsson P, Suominen A, Brown AS, Sourander A. Attention-deficit/hyperactivity disorder and risk for psychiatric and neurodevelopmental disorders in siblings. *Psychol Med* 2019; **49**: 84-91.
9. Yao S, Kuja-Halkola R, Thornton LM, et al. Familial Liability for Eating Disorders and Suicide Attempts: Evidence From a Population Registry in Sweden. *JAMA Psychiatry* 2016; **73**: 284-91.
10. Hudson JI, Javaras KN, Laird NM, VanderWeele TJ, Pope HG, Jr., Hernan MA. A structural approach to the familial coaggregation of disorders. *Epidemiology* 2008; **19**: 431-9.
11. Rom AL, Wu CS, Olsen J, Jawaheer D, Hetland ML, Mørch LS. Parental Rheumatoid Arthritis and Autism Spectrum Disorders in Offspring: A Danish Nationwide Cohort Study. *J Am Acad Child Adolesc Psychiatry* 2018; **57**: 28-32.e1.
12. Mataix-Cols D, Frans E, Perez-Vigil A, et al. A total-population multigenerational family clustering study of autoimmune diseases in obsessive-compulsive disorder and Tourette's/chronic tic disorders. *Mol Psychiatry* 2018; **23**: 1652-8.
13. Benros ME, Eaton WW, Mortensen PB. The epidemiologic evidence linking autoimmune diseases and psychosis. *Biol Psychiatry* 2014; **75**: 300-6.
14. Dalsgaard S, Waltoft BL, Leckman JF, Mortensen PB. Maternal history of autoimmune disease and later development of tourette syndrome in offspring. *J Am Acad Child Adolesc Psychiatry* 2015; **54**: 495-501 e1.
15. Benros ME, Waltoft BL, Nordentoft M, et al. Autoimmune diseases and severe infections as risk factors for mood disorders: a nationwide study. *JAMA Psychiatry* 2013; **70**: 812-20.
16. Suren P, Bakken IJ, Lie KK, et al. Differences across counties in the registered prevalence of autism, ADHD, epilepsy and cerebral palsy in Norway. *Tidsskr Nor Laegeforen* 2013; **133**: 1929-34.
17. Exarchou S, Lindstrom U, Askling J, et al. The prevalence of clinically diagnosed ankylosing spondylitis and its clinical manifestations: a nationwide register study. *Arthritis Res Ther* 2015; **17**: 118.
18. Centre for Epidemiology Swedish National Board of Health and Welfare. *The Swedish Medical Birth Register- A summary of content and quality*; 2003.
19. Ludvigsson JF, Andersson E, Ekbom A, et al. External review and validation of the Swedish national inpatient register. *BMC Public Health* 2011; **11**: 450.
